# How much of the unemployment effect on mental health is due to income? Mediation analysis in UK data

**DOI:** 10.1093/eurpub/ckac129.341

**Published:** 2022-10-25

**Authors:** RM Thomson, D Kopasker, A Leyland, A Pearce, SV Katikireddi

**Affiliations:** MRC/CSO Social and Public Health Sciences Unit, Glasgow, UK

## Abstract

**Background:**

Employment and income are important determinants of mental health (MH), but the extent to which unemployment effects are mediated by reduced income is unclear. We estimated the total effect (TE) of unemployment on MH and the controlled direct effect (CDE) not acting via income.

**Methods:**

We studied adults 25-64y from nine waves of the representative UK Household Longitudinal Study (n = 45,497/obs=202,297). Unemployment was defined as not being in paid employment; common mental disorder (CMD) was defined as a General Health Questionnaire-12 score ≥4. We conducted causal mediation analysis using inverse probability of treatment weights to estimate odds ratios (OR) and absolute differences for the effects of unemployment on CMD as measured in the same sweep, before (TE) and after (CDE) blocking the income pathway. The percentage mediated by income was 100*(TE-CDE)/TE, with standard errors calculated via bootstrapping. Multiple imputation addressed missingness.

**Results:**

The TE of unemployment on short-term CMD risk was OR: 1.66 (95% CI 1.57-1.76), with 7.09% (6.21-7.97) absolute difference in prevalence; equivalent CDEs were OR 1.55 (1.46-1.66) and 6.08% (5.13-7.03). Income mediated 14.22% (8.04-20.40) of the TE. Percentage mediation was higher for job losses (15.10% [6.81-23.39]) than job gains (8.77% [0.36-17.19]). Mediation by income was lowest for those aged 25-40y (7.99% [-2.57, 18.51]) and those in poverty (2.63% [-2.22, 7.49]).

**Conclusions:**

In the UK, a high proportion of the short-term effect of unemployment on MH is not explained by income, particularly for those who are younger or already living in poverty. Population attributable fractions suggested 16.5% of CMD burden was due to unemployment, with 13.9% directly attributable to job loss rather than resultant income changes. Further research is needed across different European countries to determine how different welfare regimes might moderate these effects, and to investigate longer-term effects.

**Key messages:**

• Unemployment has a clear detrimental effect on MH in the short-term.

• Only a small proportion of this effect appears to be mediated by income.

